# Prevalence and Correlates of Herbal Medicine Use among Women Seeking Care for Infertility in Freetown, Sierra Leone

**DOI:** 10.1155/2018/9493807

**Published:** 2018-04-22

**Authors:** Peter Bai James, Lexina Taidy-Leigh, Abdulai Jawo Bah, Joseph Sam Kanu, Jia Bainga Kangbai, Stephen Sevalie

**Affiliations:** ^1^Faculty of Pharmaceutical Sciences, College of Medicine and Allied Health Sciences, University of Sierra Leone, Freetown, Sierra Leone; ^2^Australian Research Centre in Complementary and Integrative Medicine, Faculty of Health, University of Technology Sydney, Ultimo, Sydney, NSW 2007, Australia; ^3^Faculty of Basic Medical Sciences, College of Medicine and Allied Health Sciences, University of Sierra Leone, Freetown, Sierra Leone; ^4^Center for International Health, University of Munich, Munich, Germany; ^5^Department of Environmental Health Sciences, School of Community Health Sciences, Njala University, Bo, Sierra Leone; ^6^34 Military Hospital, Freetown, Sierra Leone

## Abstract

In resource-poor countries where access to infertility care is limited, women may turn to traditional medicine to achieve motherhood. It is unknown whether Sierra Leonean women with such condition use herbal medicine. This study investigates the prevalence and factors associated with herbal medicine use among women seeking care for infertility. This was a questionnaire-based cross-sectional study conducted among women seeking care for infertility at various clinics within Freetown, Sierra Leone. Data analysis included Chi-square tests and logistic regression. Out of the 167 women that participated, 36.5% used herbal medicine for infertility treatment. Women with no formal (AOR 4.03, CL: 1.38–11.76, *p* = 0.011), primary education (AOR: 6.23, CL: 2.02–19.23, *p* = 0.001) and those that visited a traditional medicine practitioner (AOR: 20.05, CL: 2.10–192.28, *p* = 0.009) as well as women suffering from other reproductive health problems (AOR: 2.57, CL: 1.13–5.83, *p* = 0.024) were more likely to use herbal medicines. Friends and family (*n* = 57, 96.7%) were the main influencers of herbal medicine use. Only (*n* = 12) 19.7% of users disclosed their status to their healthcare provider. Over half (*n* = 32, 52.5%) could not remember the name of the herb they used*. Luffa acutangula* (*n* = 29, 100%) was the herbal medicinal plant users could recall. Herbal medicine use among women seeking care for infertility in Freetown is common. Healthcare providers should be aware of the potential dyadic use of herbal and allopathic medicines by their patients and be knowledgeable about commonly used herbal remedies as well as being proactive in communicating the potential risks and benefits associated with their use.

## 1. Introduction

Infertility is considered a social and public health problem that affects the health and wellbeing of millions of couples worldwide [1]. The World health Organization (WHO) defines infertility as the “failure to conceive after 12 months of regular unprotected sexual intercourse in the absence of known reproductive pathology [2]”. Globally, in the past two decades, the absolute number of couples affected by infertility has increased from 42.0 million in 1990 to 48.5 million in 2010 [3]. In Sub-Saharan Africa, infertility still receives less attention and is of low priority in the continent's reproductive health agenda [4, 5] despite its huge psychosocial and economic impact on individuals, families, and communities [4, 6–8]. It is believed that increased population growth due to high fertility rate in the region has masked the spotlight infertility deserves. Paradoxically, the prevalence of infertility in certain Sub-Saharan African countries is reported to be more than 30% [9].

Infertility or childlessness in most developing countries including Africa is gender biased with the female partner often cited as the cause of the problem [10, 11]. In many of these communities, women are target of psychological and physical abuse by their families and communities. Such an abuse can be in the form of marital instability, divorce, social isolation stigma, economic deprivation, and intimate partner violence [8, 12–14]. Bearing a child in Africa not only defines womanhood but also brings dignity and respect to the family as well as securing rights of property and inheritance [13]. It also serves to guarantee the continuation of the family lineage and future social insurance against poverty in a region where social security schemes during old age are uncommon [15]. Family and societal pressure to conceive and the increasing odds of reduced fertility due to aging [16], together with the inability to access high cost conventional medical therapies such as in vitro fertilization (IVF) and assisted reproductive technology (ART) [10, 17, 18], may influence a woman's decision to seek complementary or alternative health approaches such as herbal therapy in order to conceive.

Herbal medicine is the prevalent form of traditional and complementary medicine use in Sub-Saharan Africa [19–21]. Reasons for its popularity is attributed to its low cost, accessibility, alignment with patient's cultural and religious values, and perceived efficacy and safety as well as dissatisfaction with conventional healthcare [22–27]. Despite the popularity of traditional and complementary medicine, evidence of its safety and efficacy still remains inconclusive. As with most countries in Africa, traditional medicine use in Sierra Leone is common with considerable amount of the population using it to treat various health conditions such malaria, diarrhea, and respiratory infections and hypertension [20, 28–31].

Unorthodox fertility services are widespread in Africa and are often provided by traditional medicine practitioners [32]. While many studies outside of Africa have looked at traditional and complementary health approaches utilized by women seeking infertility care [33–37], only few studies across Africa have focused on this issue with relatively high use reported in these studies. A Ugandan study reported 76.2% prevalence of herbal medicine use among women seeking infertility care [38], while a Nigerian study reported that more than two-thirds of infertile couples (69%) seek care from a traditional complementary medicine practitioner [32]. Another study from Rwanda reported that 11% of the 277 women who took part in the study visited a traditional healer [39]. Even though herbal medicine use is reported to be widespread in Sierra Leone [29–31, 40], it is unknown whether women seeking infertility care use herbal medicines for their condition. It is against this background that this study was conducted to determine the prevalence and factors associated with herbal medicine use among women seeking care for infertility in Freetown, Sierra Leone.

## 2. Methodology

### 2.1. Study Design and Population

A quantitative cross-sectional study was performed among women seeking fertility care at various clinics within the municipality of Freetown, Sierra Leone. The study was done between the months of September 2016 and November 2016. Women between the ages of 18–49 years who were seeking fertility care, of various ethnic groups and religious backgrounds, were eligible to participate. Severely ill women were excluded from the study.

### 2.2. Study Setting

Area selected for this study was Western Area Urban, Freetown. Six maternity clinics providing fertility care to patients were purposefully selected. We purposefully chose the cited clinics because they are the ones providing fertility care to women in Freetown. These health facilities include Mary-Immaculate Maternity Clinic, Women's Healthcare Centre, Family Medical Care Centre, Marie-Stopes clinics at the western and eastern parts of Freetown, and 34 Military Hospital. Selection of participants from the various clinics was done through consecutive sampling and the target number in each clinic was through the proportional representation based on the attendance rate at each facility.

### 2.3. Sample Size and Determination

A target sample of 192 women was determined using the formula for sample size calculation for cross-sectional study; that is, *n* = *z*^2^*pq*/*d*^2^, where *n* is required minimum sample size, *z* is value of test statistics (1.96),* q* is probability of those not using herbs, that is, (1 − *p*),* d* is degree of accuracy or standard error (0.06), and* p* is estimated proportion of use of herbal medicine among women seeking fertility care. We assumed* p* = 76.2% based on a similar study conducted in Uganda [38].

### 2.4. Study Questionnaire

The study questionnaire was developed based on available literature from similar studies done in Uganda [38], United Kingdom [41], Turkey [37], Australia [42], and Lebanon [35]. The drafted questionnaire was pretested among 15 women having fertility issues whose data were excluded in the final analysis. Based on the feedback from this pretest, changes were made on the initial draft which was then used in the actual study. The study questionnaire comprised four (4) sections. The first section consists of the sociodemographic characteristics of the participant such as age, tribe, religion, marital status, and education level. The second section looked at the general and reproductive health status of the participant. The third section consists of questions regarding participants use of herbal medicine. The fourth and final section looked at the general perception of herbal medicine among respondents. Herbal medicines considered in this study were based on the WHO definition which includes herbs (such as leaves, flowers, fruits, seeds, stems, wood, bark, roots, or other plant parts which may be entire, fragmented, or powdered), herbal materials (such as fresh juices, gums, oils, dry powders obtained by procedures like steaming, roasting, etc.), herbal preparations (finished herbal products including powdered herbal materials or extracts, tinctures, and oils of herbal materials, and also those made in the form of beverages), and finished herbal products that contain active ingredients as parts of plants, or other plant materials or combinations [43]. Herbal medicine users were defined as women in the inclusion group that reported the use of herbal medicine for infertility for the past twelve months administered orally and/or through any other route of drug administration.

### 2.5. Data Collection

The data was collected through face-to-face interview for participants who were illiterate as well as a self-administered format for women who can read and write. The purpose of the study was explained to the patients and those who consented were interviewed. Participants were assured of their confidentiality and given the liberty to opt out of the study at any time while filling the form or being interviewed. A consent form was signed to confirm their willingness to participate in the study. To help minimize social desirability bias, data collectors were extensively trained on the rubrics of data collection process such as not being judgmental, being neutral, and avoiding asking questions that can influence participant response [44].

### 2.6. Data Analysis

Data analysis was done using SPSS Package version 24 (SPSS, Inc; Chicago). Descriptive statistics were used to calculate frequency counts and percentages for categorical variables and mean standard deviation for continuous variables. Chi-square and Fischer exact two tailed tests were used to determine the association between herbal medicine use (dependent variable) and demographic and health-related variables (covariates). In order to adjust for possible confounders and evaluate independent effects of each independent variable on the outcome variable (herbal medicine use), univariate analysis was conducted and demographic and health-related variables that show statistically significant association were then entered into multivariate logistic model. Differences were considered statistically significant if the *p* value was less than 0.05.

### 2.7. Ethical Clearance

Ethical clearance for this study was sought from the Research and Ethics Committee of COMAHS-USL.

## 3. Results

### 3.1. Sociodemographic and Health-Related Characteristics of Women Seeking Care for Infertility

Out of the 192 approached, 167 agreed to participate, giving a response rate of 89.8%. 88 participants were between the ages of 20–29 years (52.7%), 110 married (65.0%), 67 attained tertiary level of education (40.1%), and 131 employed (78.4%), and those with monthly income between 1.5 and 3 million Leones, 127 (76%), were predominant. Please see Table 1 for more details.

### 3.2. Association between Sociodemographic and Health-Related Factors and Herbal Medicine Use among Women Seeking Care for Infertility

Based on the results from data analysis, educational status (*p* < 0.001) and monthly income (*p* < 0.001) and those without other reproductive health problems apart from infertility (*p* = 0.017), those who had previously given birth (*p* = 0.017), those who faced barriers to accessing conventional fertility care (*p* < 0.001), and those who did not visit a traditional medicine practitioner (*p* < 0.001) were shown to have statistically significant association with the use of herbal medicine as seen in Table 2.

### 3.3. Predictors of Herbal Medicine Use among Women Seeking Care for Infertility

Table 3 presents univariate and multivariate regression analysis of possible predictors of herbal medicine use among women seeking fertility care. Women who had no formal (AOR 4.03, CL 1.38–11.76, *p* = 0.011) or primary education (AOR: 6.23, CL: 2.02–19.23, *p* = 0.001) were 4 and 6 times more likely to use herbal medicines than those that attained tertiary education, respectively. Also, women who visited a traditional medicine practitioner (AOR: 20.05, CL: 2.10–192.28, *p* = 0.009) were 20 times more likely to use herbal medicine than those who did not visit a traditional medicine practitioner. In addition, women suffering from other reproductive health problems were almost three times more likely to use herbal medicine than those who did not suffer from other reproductive health problems (AOR: 2.57, CL: 1.13–5.83, *p* = 0.024).

### 3.4. Pattern of Herbal Medicine Use among Women Seeking Care for Infertility

Based on the analyzed data in Table 4, 36.5% of the total number of participants (*n* = 167) have used or are currently using herbal medicine for their condition, with the majority (96.7%) of which doing so due to recommendation by friends and family. Only 47.5% of participants who used herbal medicine knew the name of the product used and Rabena* (Luffa acutangula)* was cited as the herbal medicine used. Route of administration was oral (100%). 11.5% experienced side effects, majority of which was amenorrhea (42.8%). A fifth (19.7%) of participants who used herbal medicine disclosed their status to their healthcare provider. Reasons for nondisclosure to healthcare provider were because the doctor did not ask (51.0%) or participants did not think it was necessary (49%).

### 3.5. Perception of Herbal Medicine Use among Women Seeking Care for Infertility (*n* = 167)

Only 1.2% of the total number of participants (*n* = 167) agreed that herbal medicines are effective for their condition. 53.3% disagreed that herbal medicines are safer than western medicines for infertility and 46.7% were not sure. Almost half (46.1%) agreed that herbal medicines are natural. About 26.3% also agreed that herbal medicines are beneficial when recommended by healthcare provider and only 1.2% agreed that it is beneficial when recommended by traditional medicine practitioner or herbalist. Only 3.6% of the total number of participants agreed that herbal medicine should be integrated into the mainstream healthcare system as in Table 5.

## 4. Discussion

This study presents the first empirical findings in Sierra Leone on traditional medicine use among women seeking conventional fertility treatment. Our study highlights key findings that are worth discussing. First, the use of herbal medicine is common (36.5%) among women undergoing biomedical infertility care. This prevalence of herbal medicine use is lower than similar studies conducted in Uganda [38] and Lebanon [35] but higher than studies conducted in USA [36], Turkey [37], Jordan [33], Australia [42], and Rwanda [39]. The difference in utilization rate observed with other countries may be partly due to variation in the availability and access to conventional healthcare and the sociocultural difference on how traditional, complementary, and alternative medicine use is perceived as well as the heterogeneity in the study design and definition of TCAM therapy used [45, 46]. Considering the widespread use of herbal medicine among the Sierra Leonean populace [47, 48], and the fact that women in Africa are under enormous pressure to conceive [5, 49], the search for answers to their predicament goes beyond seeking conventional care to also include alternative medical care in the form of herbal medicine [50]. In certain instances, TCAM is considered as the first-choice therapy when the cause of infertility is perceived to be nonmedical or nonconventional treatment is considered to be much more effective than conventional therapies [51]. Also, the high cost of conventional therapies such as the use of assisted reproductive technologies (ART) [18] may be a push factor that drives women to consider alternative health approaches such as herbal medicine as their preferred healthcare choice for their condition.

In our study women cited friends and family members as key influencers in their decision to use herbal medicine. In Africa, giving birth to a child defines womanhood and brings public honour and respect to both families [13]. Also, decision-making in Africa regarding reproductive health is often influenced by older family members [52]. As such, the decision to use complementary and alternative medicine is often a family rather than an individual decision. Besides its biomedical cause, infertility, in the African society, is often associated with supernatural and spiritual causes [12, 51] which makes the use of faith healing or a visit to a traditional healer a first-choice health-seeking behavior [12, 32]. Thus, the use of herbal medicine in such circumstances goes beyond its medicinal effect but also for the perceived spiritual, ritualistic, or supernatural power that it possess as dictated by tradition and culture [53]. As such, biomedical fertility care providers should be mindful of these social and cultural dynamics that may influence infertile women health-seeking behavior and routinely enquire about infertile women use of TCAM modalities especially herbal medicine use during consultation. This will create a platform to discuss the risk and benefits of herbal therapy use by their patients and provide appropriate advice. The effectiveness of such discussion requires healthcare providers to be knowledgeable about the commonly used herbal remedies for infertility and their ability to approach such discussion without any prejudice against their patients.

Our finding that herbal medicine users were more likely to be less educated is in line with a similar study conducted in Lebanon [35] but is in contrast with similar studies in Uganda [38] and the UK [41] in which less educated women were less likely to use herbal medicine as well as in the United States in which no significant difference was observed [36]. The high use of herbal medicine among less educated women may be due to less exposure and less knowledge about risk and benefits of herbal medicine use compared to their highly educated counterparts who are likely to make well informed choices. For the less educated women, their decision to use herbal medicine may have been based on the recommendation from their trusted peers. This speculation is supported by our finding that the decision to use herbal medicine was mainly influenced by the recommendation of friends and family. It may also be due to economic status of herbal medicine users in which those that are less educated are likely to be low income earners and as such may likely seek low cost therapeutic options like herbal remedies. Such proposed explanation seems to be supported by our findings in Tables 2 and 3 in which women with low monthly income and those that are less educated were likely to be associated with herbal medicine use. Similar findings were reported by Addo in Ghana [54]. Our study also revealed that herbal medicine use was more common among those who visited traditional medicine practitioners than those who did not. This is expected due to the fact that traditional medicine practitioners are likely to recommend the use of herbs since it is the mainstay of their therapeutic intervention. We also observed in our study that women with other reproductive health problems were more likely to use herbal medicine compared to those without other reproductive health problems. This means that the use of herbal therapy in this study is not entirely directed at enhancing fertility or treating infertility but treating other reproductive health conditions that may limit women's chance to conceive.

The concurrent use of herbal and allopathic medicine in our study poses a threat to patient safety and fertility treatment outcome due to adverse effects and therapeutic failure as a result of herbal-drug interactions and/or herbal medicine contamination [55, 56]. Patient safety and treatment outcome are further hampered in that the most cited herbal medicine Rabena* (Luffa acutangula)* does exhibit abortifacient effect [57, 58] which can potentially prevent an infertile woman's quest to give birth. In addition, the risk of adverse effect increases with the fact that the choice to use herbal medicine was greatly influenced by people with low level of knowledge about the safety and efficacy of herbal remedies which is in line with findings from Lebanon [35]. This further strengthens the need for fertility care providers to be knowledgeable about common herbal therapies and always take comprehensive medication history of their patients with the aim of detecting potential adverse effects which could undermine the outcome of infertility care being provided. Also, public education and counselling of patients are needed since our study shows a gap in awareness about herbal medicine among users.

We observed in our study that there was a low disclosure rate of herbal medicine use among users. This is in line with similar studies in Uganda [38] and Lebanon [5]. The reasons for nondisclosure were that healthcare providers failed to ask and the thought that it not necessary to divulge such information which resonates with the current literature on the nature of physician-patient communication regarding herbal medicine use in Africa [19, 59]. Other reasons for nondisclosure cited in the literature include fear of health provider's reaction that can potentially undermine care and perceived lack of support and understanding from conventional healthcare providers [60–62]. Effective communication between patients and providers is essential to achieving the desired goal of infertility care, the absence of which can negatively affect patient's treatment outcome. Therefore, healthcare providers should be aware of this and always initiate discussion surrounding use of alternative medical care with their patients that is free of prejudice but based on mutual respect. This will encourage patient to freely discuss with their providers wide varieties of issues including traditional medicine use.

## 5. Study Limitations and Strengths

The following limitations need to be considered when interpreting the results of our study. Our results did not represent the views of infertile women in Sierra Leone since this study was only done in the city. Follow-up studies conducted nationally or in other areas of the Sierra Leone are needed to confirm the consistency of our findings. Also, qualitative studies are required to deeply explore this topic in order to fully understand how nonconventional health approaches interact or interface with infertility care in Sierra Leone. In addition, since interviews were conducted in a conventional healthcare setting, the reported prevalence of herbal medicine use might be an underestimation of the actual utilization rate as there is potential bias towards biomedical care among participants. Nevertheless, our study presents the first empirical evidence of herbal medicine use among women seeking infertility care in Sierra Leone and will help influence policy decisions and mode of practice regarding infertility care in Sierra Leone. For example, our findings will help fertility care providers to identify those who are likely users of herbal medicine. The results of our study also emphasize the need for healthcare providers to routinely include the discussion on herbal medicine use during consultation with their patients and advice appropriately with the aim of promoting a favorable health outcome for their patients. In terms of policy, our study provides evidence for public education and counselling of women on the risks and benefits associated with the use of complementary and alternative health approaches for infertility. Since this is the first ever study on this topic in Sierra Leone, our findings add to the scanty literature surrounding alternative or complementary healthcare and infertility in Africa and Sierra Leone in particular and will help provide the basis for further studies to be conducted in Sierra Leone and other African countries.

## 6. Conclusion

The use of herbal medicine among women seeking care for infertility in Freetown, Sierra Leone, is common. Health professionals providing fertility care should be mindful of the pluralistic health-seeking behavior of patients under their care. It is also essential for them to be knowledgeable about the common herbal medicines used for infertility treatment and to routinely initiate dialogue with patients on their risks and benefits.

## Figures and Tables

**Table 1:** Sociodemographic and health-related characteristics.

Characteristics	Variables	*N* (%)
Age group	20–29 years	88 (52.7)
	30–39 years	78 (46.7)
	40–49 years	1 (0.6)

Tribe	Mende	44 (26.3)
	Temne	39 (23.4)
	Fullah	12 (7.2)
	Others	72 (43.1)

Marital status	Single	10 (6.0)
	Married	110 (65.0)
	Cohabitate	45 (26.9)
	Separated	2 (1.2)

If married, type of marriage, *n* = 110	Monogamous	101 (91.0)
	Polygamous	10 (9.0)

If polygamous, number of wives, *n* = 10	Two wives	8 (80.0)
	Three wives	2 (20.0)

Duration of relationship	1–5 yrs	105 (61.7)
	6–10 yrs	55 (32.9)
	>10 yrs	9 (5.4)

Highest level of education attained	No formal education	38 (22.8)
	Primary	26 (15.6)
	Secondary	36 (21.6)
	Tertiary	67 (40.1)

Religion	Christianity	80 (47.9)
	Islam	87 (52.1)

Provider of household income	Self	7 (4.2)
	Partner	47 (28.1)
	Both	109 (65.3)
	Others (parent or in-laws etc.)	4 (2.4)

Employment status	Employed	131 (78.4)
	Unemployed	36 (21.6)

Monthly income	<1 million Leones	40 (24.0)
	1–3.5 million Leones	127 (76.0)

Distance from health facility	<5 km	94 (56.3)
	5–10 km	69 (41.3)
	>10 km	4 (2.4)

Presence of health problem	Yes	24 (14.4)
	No	143 (85.6)

If yes, what type, *n* = 24	Peptic ulcer	8 (33.3)
	Malaria	6 (25.0)
	Typhoid fever	4 (16.7)
	Asthma	3 (12.5)
	Diabetes	2 (8.3)
	Sickle cell	1 (4.2)
	Hypertension	1 (4.2)

Suffering from reproductive health problems	Yes	57 (34.1)
	No	110 (65.9)

If yes, what type, *n* = 57	Miscarriage	29 (50.9)
	Vaginal infection	15 (26.3)
	Sexual transmitted infection	9 (15.8)
	Fibroid	2 (3.5)
	Bleeding	1 (1.7)
	Painful menstruation	1 (1.7)

If yes, seek help, *n* = 57	Yes	52 (91.2)
	No	5 (8.8)

If yes, where did you seek help, *n* = 57	Health facility	57 (100)

Previously given birth	Yes	81 (48.5)
	No	86 (51.5)

Mean number of children *n* = 81		1.5 (0.6)^*∗*^

Change of partner	Yes	15 (9.0)
	No	152 (91.0)

Suffering from emotional torture from partner or immediate family	Rejection	11 (6.6)
	Violence	5 (3.0)
	Stigma	6 (3.6)
	None	145 (86.8)

Cause of infertility	Witchcraft	1 (0.6)
	Curse/spell	1 (0.6)
	Medical causes	165 (98.8)

Barriers to accessing fertility care	Yes	11 (6.6)
	No	156 (93.4)

If yes, what type of barriers, *n* = 23	Financial	15 (65.2)
	Distance	7 (30.4)
	Family	1 (4.4)

Duration of infertility (months)		12.9 (11.3)^*∗*^

Visit to traditional medicine practitioner	Yes	11 (6.6)
	No	156 (93.4)

^*∗*^Mean (standard deviation). $1 = SLL7500 at the time of conducting the study.

**Table 2:** Association between sociodemographic and health-related factors and herbal medicine use among women seeking fertility care.

Characteristics	Variables	Users *n*	Nonusers *n*	*p* value
Age group	<30 years	27	61	0.098
	≥30 years	34	45	

Religion	Christianity	28	52	0.694
	Islam	33	54	

Marital status	Single/separated	3	9	0.389
	Married/cohabitate	58	97	

Type of marriage	Monogamous	33	68	0.639
	Polygamous	4	6	

Change of partner	No	54	98	0.393
	Yes	7	8	

Duration of relationship	1–5 yrs	32	71	0.069
	6–10 yrs	23	32	
	>10 yrs	6	3	

Tribe	Mende	15	29	0.470
	Temne	16	23	
	Fullah	2	10	
	Others	28	44	

Educational status	No formal education	22	**16**	<0.001
	Primary	16	10	
	Secondary	11	25	
	Tertiary	12	55	

Employment status	Employed	48	83	0.953
	Unemployed	13	23	

Monthly income	<1 million Leones	24	16	<0.001
	1–3.5 million Leones	37	90	

Partner with child/children from previous relationship	Yes	26	30	0.066
	No	33	71	

Household income provider	Self	3	4	0.383
	Partner	20	27	
	Both	38	71	
	Others	0	4	

Duration of infertility	0–24 months	52	100	0.048
	>24 months	9	6	

Distance from health facility	<5 km	29	65	0.084
	5–10 km	29	40	
	>10 km	3	1	

Cause of infertility	Supernatural/curse	2	0	0.132
	Medical	59	106	

Presence of health problems	Yes	8	16	0.725
	No	53	90	

Presence of other reproductive health conditions other than infertility	Yes	28	29	0.017
	No	33	76	

Previously given birth	Yes	37	44	0.017
	No	24	62	

Reported emotional torture	Yes	10	12	0.351
	No	51	94	

Barrier to accessing conventional fertility care	Yes	34	30	<0.001
	No	27	76	

Visit to traditional medicine practitioner	Yes	10	1	<0.001
	No	51	105	

**Table 3:** Predictors of herbal medicine use among women seeking care for infertility.

Characteristics	Variables	COR (95% CL)	*p* value	AOR (95% CL)	*p* value
Age group	<30 years	1			
	≥30 years	1.70 (0.90–3.22)	0.099	-	

Religion	Islam	1	0.694		
	Christianity	0.88 (0.47–1.66)		-	

Marital status	Single/separated	1	0.395		
	Married/cohabitate	1.79 (0.47–6.90)		-	

Type of marriage	Polygamous	1	0.640		
	Monogamous	0.73 (0.19–2.76)		-	

Change of partner	No	1	0.396		
	Yes	1.59 (0.55–4.62)		-	

Duration of relationship	>10 yrs	1		1	
	1–5 yrs	0.23 (0.05–0.96)	0.044	0.27 (0.04–1.59)	0.147
	6–10 yrs	0.36 (0.08–1.59)	0.177	0.31 (0.06–1.68)	0.173

Tribe	Others	1			
	Mende	0.81 (0.37–1.78)	0.604	-	
	Temne	1.09 (0.49–2.42)	0.826	-	
	Fullah	0.31 (0.06–1.54)	0.154	-	

Educational status	Tertiary	1		1	
	Nonformal	6.30 (2.57–15.46)	<0.001	4.03 (1.38–11.76)	0.011
	Primary	7.33 (2.68–20.08)	<0.001	6.23 (2.02–19.23)	0.001
	Secondary	2.01 (0.78–5.19)	0.146	1.90 (0.62–5.85)	0.262

Employment status	Unemployed	1	0.953		
	Employed	1.02 (0.47–2.20)		-	

Partner with child/children from previous relationship	No	1	0.068		
	Yes	1.87 (0.96–3.64)			

Household income provider	Self	1			
	Partner	0.99 (0.19–4.92)	0.988	-	
	Both	0.71 (0.15–3.36)	0.669	-	
	Others	0.00 (0.00)	0.999	-	

Monthly income	1–3.5 million Leones	1	0.001	1	
	<1 million	3.65 (1.74–7.64)		1.95 (0.70–5.42)	0.202

Distance from health facility	>10 km	1		-	
	<5 km	0.15 (0.02–1.49)	0.105	-	
	5–10 km	0.24 (0.02–2.44)	0.229	-	

Presence of health problems	No	1			
	Yes	0.85 (0.34–2.12)	0.726	-	

Suffering from other reproductive health problems	No	1		1	
	Yes	2.22 (1.15–4.31)	0.018	2.57 (1.13–5.83)	0.024

Reported emotional torture	No	1	0.353	-	
	Yes	1.54 (0.62–3.80)		-	

Barriers to accessing fertility care	No	1		1	
	Yes	3.19 (1.65–6.16)	0.001	1.46 (0.61–3.49)	0.400

Visited a traditional medicine practitioner	No	1		1	
	Yes	20.59 (2.57–165.24)	0.004	20.05 (2.10–192.28)	0.009

Duration of infertility	>24 months	1			
	0–24 months	0.35 (0.12–1.03)	0.056	-	

Previously pregnant	No	1		1	
	Yes	2.17 (1.14–4.13)	0.018	2.32 (0.93–5.82)	0.072

**Table 4:** Pattern of herbal medicine use among women seeking infertility care.

Characteristics	Variable	*N* (%)
The use of herbal medicine to treat your condition in the past twelve months	Yes	61 (36.5)
	No	106 (63.5)

Reason for using herbal medicine *n* = 61	Recommended by friends and family	57 (96.7)
	Others (recommended by herbal medicine seller)	2 (3.3)

Awareness of the name of the herbal medicine used, *n* = 61	Yes	29 (47.5)
	No	32 (52.5)

If yes, name of herbal medicine, *n* = 29	Rabena *(Luffa acutangula)*	29 (100)

Route of administration of herbal medicine, *n* = 61	Orally	61 (100)

If yes, was it beneficial? *n* = 61	No	61 (100)

Experienced side effects, *n* = 61	Yes	7 (11.5)
	No	54 (88.5)

If yes, type of side effect, *n* = 7	Amenorrhea	3 (42.8)
	Pruritus	2 (28.6)
	Rash	2 (28.6)

Disclosure to healthcare provider	Yes	12 (19.7)
	No	49 (80.3)

If no, reason for nondisclosure, *n* = 49	Health provider did not ask	25 (51.0)
	Thought it was not necessary	24 (49.0)

**Table 5:** Perception of herbal medicine use among women seeking care for infertility (*n* = 167).

Statements	Agree *n* (%)	Disagree *n* (%)	Not sure *n* (%)
Herbal medicines are effective for your condition	2 (1.2)	87 (52.1)	78 (46.7)
Herbal medicines are safer than western medicines for your condition	0 (0)	89 (53.3)	78 (46.7)
Herbal medicines are natural	77 (46.1)	9 (5.4)	81 (48.5)
Herbal medicines are beneficial if recommended by healthcare provider	44 (26.3)	13 (7.8)	110 (65.9)
Herbal medicines are beneficial if recommended by traditional medicine practitioner or herbalist	2 (1.2)	46 (27.5)	119 (71.3)
Herbal medicines should be integrated into the mainstream healthcare system	6 (3.6)	18 (10.8)	143 (85.6)

## Abbreviations

TCAM: Traditional complementary and alternative medicine
COMAHS-USL: College of Medicine and Allied Health Sciences, University of Sierra Leone.
